# Erosive pustulöse Dermatose der Unterschenkel (EPDL): Eine selten diagnostizierte neutrophile Dermatose älterer Menschen

**DOI:** 10.1111/ddg.70071

**Published:** 2026-07-07

**Authors:** Joachim Dissemond, Jan Kottner, Jan‐Malte Placke, Cornelia Erfurt‐Berge

**Affiliations:** ^1^ Klinik und Poliklinik für Dermatologie Venerologie und Allergologie Universitätsklinikum Duisburg‐Essen, Essen; ^2^ Charité‐Universitätsmedizin Berlin Institut für klinische Pflegewissenschaft, Berlin; ^3^ Hautklinik Universitätsklinikum Erlangen, Erlangen

**Keywords:** Atrophie, chronische venöse Insuffizienz, Kompressionstherapie, Neutrophile Dermatose, Pusteln, atrophy, chronic venous insufficiency, compression therapy, neutrophilic dermatosis, pustules

## Abstract

Die erosive pustulöse Dermatose der Unterschenkel (EPDL) ist eine selten diagnostizierte, chronische entzündliche Hauterkrankung, die überwiegend bei älteren Menschen auftritt. Prädisponierende Faktoren sind unter anderem Hautatrophie, chronische venöse Insuffizienz und Traumata. Auch wenn die Pathogenese der EPDL bislang nicht abschließend geklärt wurde, wird diskutiert, dass es sich um eine neutrophile Dermatose handelt, bei der es durch exogene Provokationsfaktoren zu einer immunologischen Fehlregulation mit lokaler Hautschädigung kommt. Klinisch manifestiert sich die EPDL mit oberflächlichen, sterilen Pusteln aus denen sich scharf begrenzte Erosionen entwickeln. Die Prädilektionsstellen sind das mittlere Drittel der Unterschenkelstreckseiten. Es handelt sich um eine Ausschlussdiagnose, was die diagnostische Abgrenzung erschwert. Therapeutisch werden hochpotente topische Glukokortikoide mit hohem therapeutischen Index (TIX) sowie Calcineurin‐Inhibitoren eingesetzt. Systemische immunmodulierende Therapien sind ausschließlich bei therapierefraktären Verläufen indiziert. Zudem ist die Optimierung der Wundbehandlung wichtig. Langfristig stehen Edukation und Hautpflege im Vordergrund der komplexen und oft langwierigen Behandlung. Aufgrund des chronisch‐rezidivierenden Verlaufs und der Gefahr sekundärer Ulzerationen sowie Superinfektionen sind frühzeitige Diagnose und individuelle Therapieplanung wichtig. Die interdisziplinäre und interprofessionelle Zusammenarbeit kann zur Verbesserung der Lebensqualität der Betroffenen und zur Reduktion des Risikos von Komplikationen entscheidend beitragen.

## EINLEITUNG

Die erosive pustulöse Dermatose des Capillitiums (*erosive pustular dermatosis of the scalp*, EPDS) wurde erstmalig 1977 von Burton beschrieben.^1^ Lanigan und Cotteril publizierten 1987 eine klinisch ähnliche Variante an den Unterschenkeln, die vorwiegend bei älteren Menschen in atrophischer und/oder aktinisch geschädigter Haut auftritt.^2^ Diese als erosive pustulöse Dermatose der Unterschenkel (*erosive pustular dermatosis of the leg*, EPDL) bezeichnete Dermatose wurde bislang in der wissenschaftlichen Literatur nur selten thematisiert. Häufig wird die Krankheit daher übersehen oder falsch diagnostiziert, weshalb verlässliche epidemiologische Daten zu Prävalenz und Inzidenz fehlen.^3^

## KLINIK

**ABBILDUNG 1:**
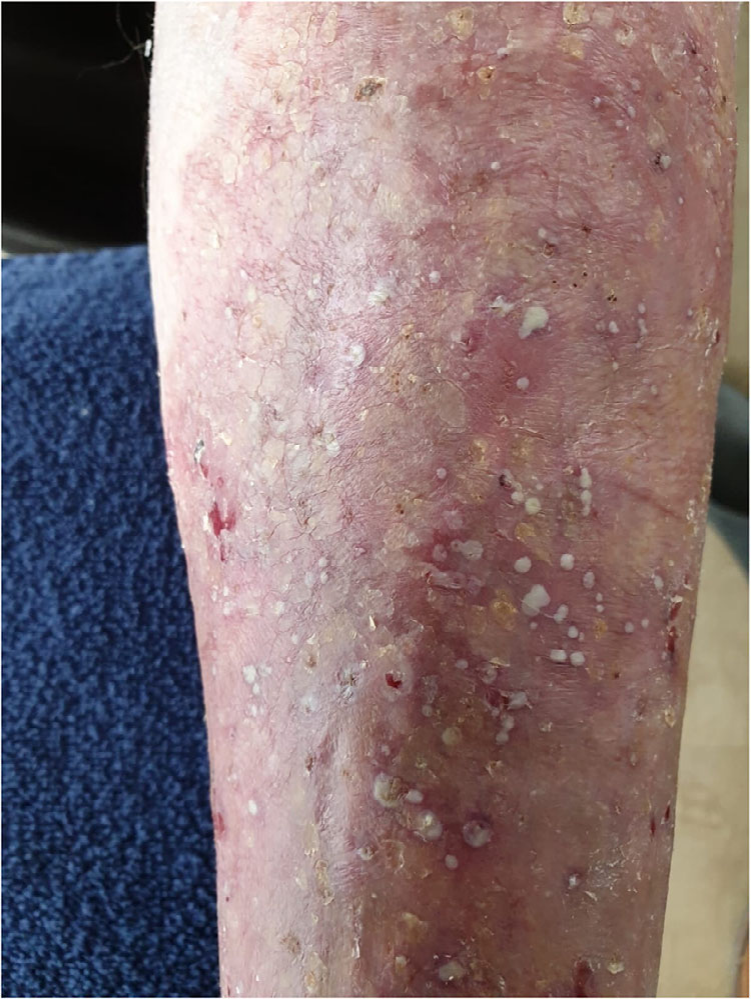
Sterile Pusteln bei einem Patienten mit CVI und Kompressionstherapie. Im weiteren Verlauf wurde die Diagnose EPDL gestellt.

**ABBILDUNG 2:**
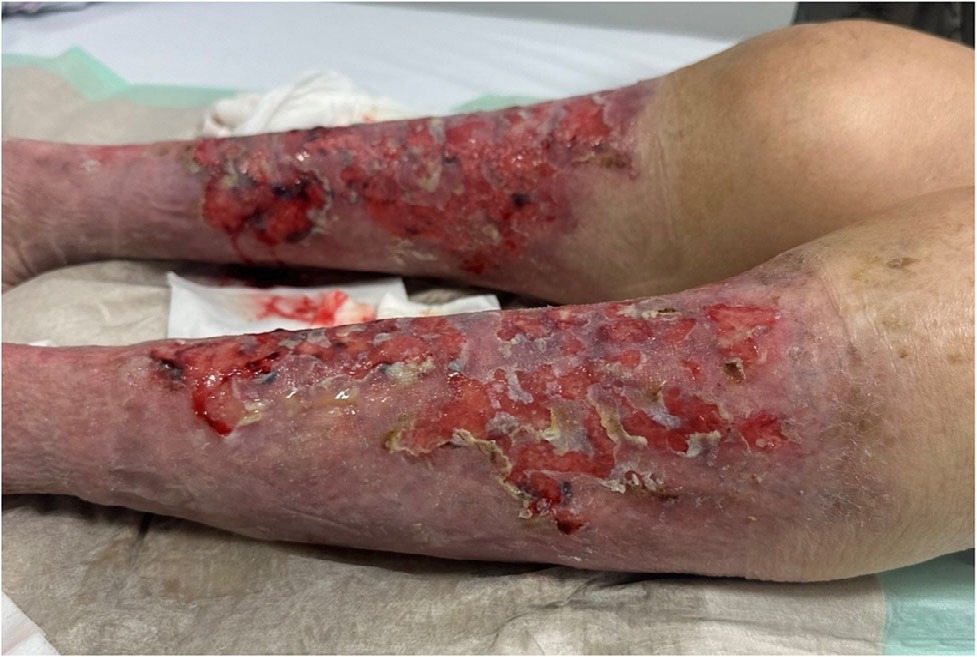
Die Begrenzung der akut aufgetretenen Erosionen bei EPDL ist scharf und serpiginös. Am Rand sieht man oft eine colleretteartige Schuppung.

**ABBILDUNG 3:**
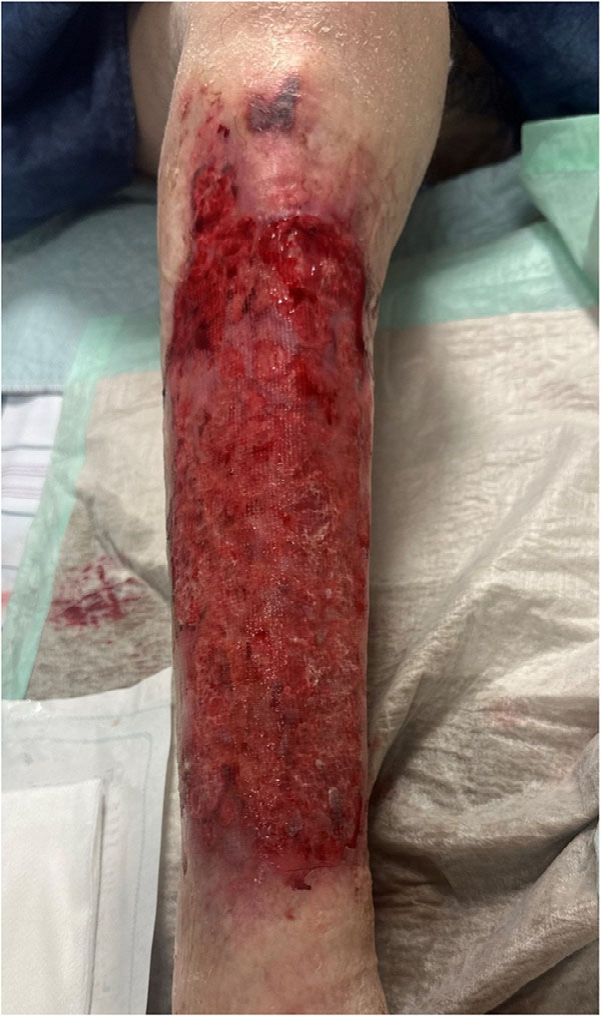
Die persistierenden Erosionen sind scharf auf das Areal mit dem Wundverband begrenzt.

Die Hautveränderungen beginnen typischerweise mit multiplen oberflächlichen, wenige Millimeter großen Pusteln (Abbildung 1) aus denen sich rasch Erosionen mit serpiginösem Rand und teils colleretteartiger Schuppung entwickeln (Abbildung 2). Im Verlauf persistiert dann eine scharf begrenzte Erosion (Abbildung 3). Auf den Erosionen können sich sekundär bakterielle und gelegentlich auch mykotische Superinfektionen und Krusten ausbilden,^4^, ^5^ auch wenn die Pusteln primär steril sind. Die Hautveränderungen treten bevorzugt ventral oder anteromedial an den Unterschenkeln auf und sind häufig beidseits lokalisiert. Die Betroffenen berichten meist über Juckreiz, Brennen und leichte Schmerzen.^4^, ^6^

## DERMATOPATHOLOGIE

**ABBILDUNG 4:**
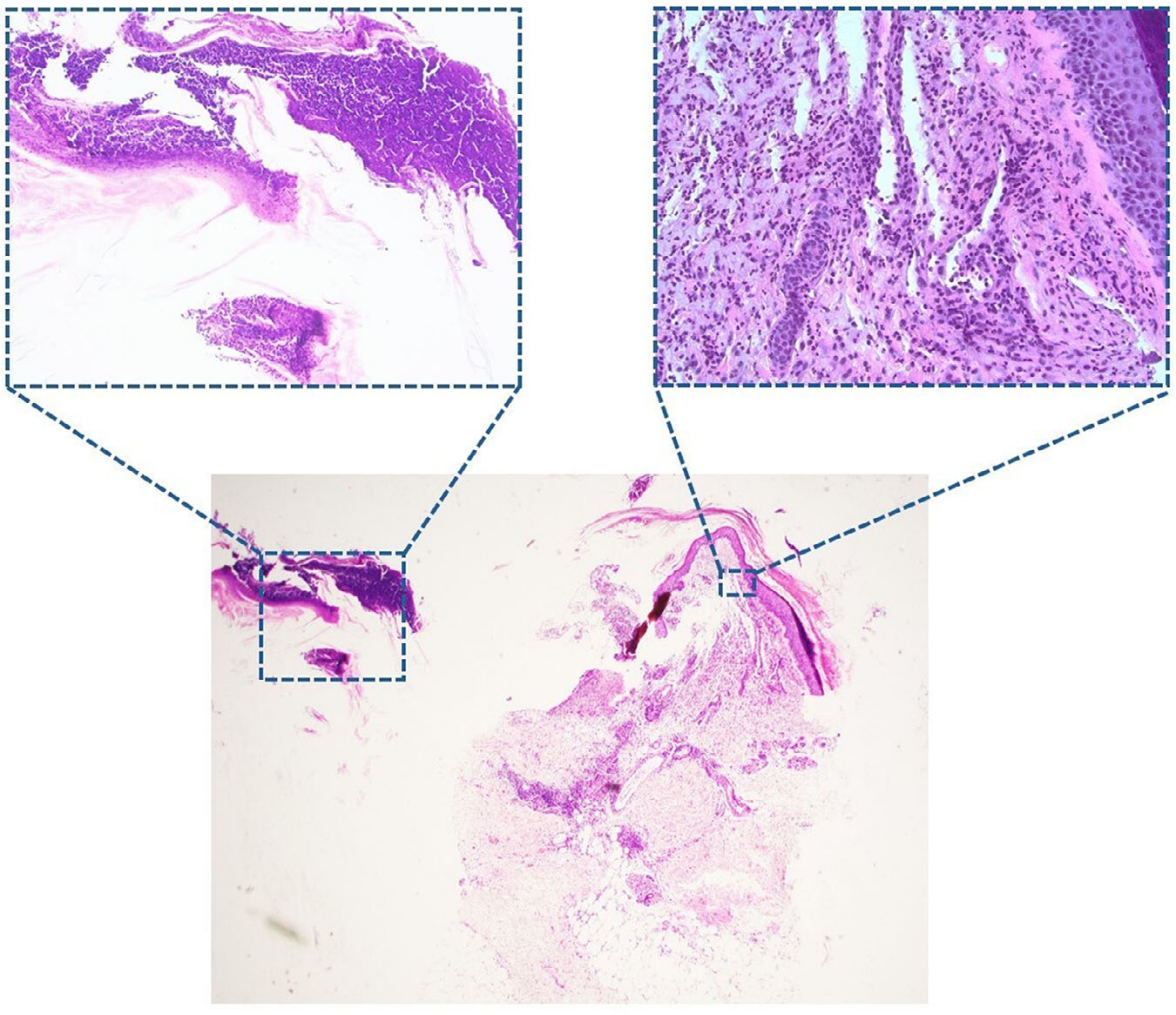
In der histopathologischen Übersichtsaufnahme imponieren Hyperparakeratose und neutrophile Granulozyten im Stratum corneum. In der oberen Dermis sind ein Ödem sowie eine gemischtzellige, neutrophilenreiche Entzündung erkennbar. Die Vergrößerung links oben zeigt das Stratum corneum mit zahlreichen neutrophilen Granulozyten, während die Vergrößerung rechts das dichte, neutrophilenreiche Entzündungsinfiltrat in der Dermis verdeutlicht (Hämatoxylin‐Eosin Färbung).

Bei unklarer Diagnose sollte eine Biopsie zur histopathologischen Abklärung erfolgen. Histopathologisch zeigen sich typischerweise sub‐ oder intracorneale Pusteln mit oberflächlicher gemischtzelliger Infiltration durch neutrophile Granulozyten und Plasmazellen sowie Atrophie der Epidermis (Abbildung 4).^5^, ^7^

Da auch das histopathologische Bild unspezifisch ist, wird die EPDL durch Ausschluss anderer Krankheiten diagnostiziert.^3^ Somit müssen die relevanten Differentialdiagnosen bekannt sein und ausgeschlossen werden (Tabelle 1).

**TABELLE 1:** Klinisch relevante Differenzialdiagnosen der EPDL.

Akute lokalisierte exanthematische Pustulose (ALEP) Bullöse Autoimmundermatosen, beispielsweise bullöses Pemphigoid Kontaktdermatitis Psoriasis pustulosa Pyoderma gangraenosum Pyodermien, wie Impetigo contagiosa, Erysipel Stauungsdermatitis

## PATHOGENESE

Die genaue Ätiopathogenese der EPDL ist bislang unklar. Beschrieben wurden verschiedene prädisponierende lokale und systemische Faktoren (Tabelle 2). In Analogie zur EPDS kann auch eine Assoziation mit Medikamenten wie Inhibitoren der EGFR (*Epidermal Growth Factor Receptor*)‐Tyrosinkinase diskutiert werden.^8^ Es wurde beschrieben, dass die EPDL, ähnlich wie andere neutrophile Dermatosen, durch die Freisetzung von DAMPs (*Damage‐Associated Molecular Patterns*) infolge von Traumata oder PAMPs (*Pathogen‐Associated Molecular Patterns*) durch Mikroorganismen ausgelöst werden kann. Hier wird eine Dysregulation des angeborenen Immunsystems mit abnormer Aktivierung neutrophiler Granulozyten induziert.^9^, ^10^ Es sind aber auch Verläufe mit spontaner Manifestation beschrieben worden.^11^

**TABELLE 2:** Prädisponierende Faktoren der EPDL.

Aktinisch geschädigte Haut Atopische Diathese Chronische venöse Insuffizienz Diabetes mellitus Ekzeme Hautatrophie, beispielsweise durch Glukokortikoide oder ionisierende Strahlung Hohes Lebensalter Malnutrition, beispielsweise Zinkmangel Mazeration insbesondere unter Wundverbänden Ödeme Traumata, etwa durch Verbände, Pflegeprodukte oder Stürze Xerosis cutis

## THERAPIE

Erfolgreiche Behandlungen basieren auf der topischen Anwendung hochpotenter Glukokortikoide.^2^, ^7^ Wegen der oft ausgeprägten Atrophie der Haut sollten möglichst ausschließlich topische Glukokortikoide mit hohem therapeutischen Index (TIX) wie Mometasonfuroat, Methylprenisolonaceponat oder Prednicarbat mit einem TIX von jeweils 2,0 verwendet werden.^12^ Gerade bei langfristiger Lokaltherapie können auch die Calcineurin‐Inhibitoren Tacrolimus oder Pimecrolimus eingesetzt werden, damit ein *Rebound*‐Phänomen nach dem Absetzen der Glukokortikoide vermieden werden kann.^13^ Aus pathophysiologischer Sicht könnten zukünftig auch topische Januskinase‐Inhibitoren (JAKi) wie Ruxolitinib, Delgocitinib ohne atrophogenes Potenzial eingesetzt werden.^14^ Allerdings fehlen hierzu bislang klinische Untersuchungen. Bei der Auswahl der individuell geeigneten Präparate sollten zwar die galenischen Grundsätze berücksichtigt werden, es ist aber auch darauf zu achten, dass die Externa möglichst atraumatisch aufgetragen werden. Die antiseptische Behandlung (beispielsweise mit Polihexanid) ist bei bakterieller Superinfektion sinnvoll.^15^ Die systemische immunmodulierende Therapie mit Glukokortikoiden oder Dapson sollte sehr ausgeprägten und therapierefraktären Verläufen vorbehalten bleiben (Tabelle 3).

**TABELLE 3:** Therapiealgorithmus der EPDL.

Therapie	Wirkstoff	Anmerkungen
**1. Wahl** **(topisch)**	Hochpotente Glukokortikoide Momethasonfuroat Methylprednisolonaceponat Prednicarbat	TIX ≥ 2,0 empfohlen
**Langzeittherapie**	Calcineurin‐Inhibitoren Tacrolimus Pimecrolimus	Vermeidung des Rebound‐Phänomens nach Glukokortikoiden
**Zukünftige Optionen** **(experimentell)**	Topische Januskinase‐Inhibitoren (JAKi) Ruxolitinib Delgocitinib	Noch keine ausreichenden klinischen Daten
**Superinfektion**	Antiseptika Polihexanid Octenidin	Begleitende Therapie bei mikrobieller Superinfektion
**Systemische Therapie**	Glukokortikoide (Prednison/Prednisolon) Dapson	Bei sehr schweren und/oder therapierefraktären Verläufen

Es ist wichtig, bekannte Risikofaktoren so weit wie möglich zu meiden. Ein praktisches Problem ergibt sich bei Patienten mit chronischer venöser Insuffizienz (CVI) und mechanischer Reizung durch die Kompressionstherapie. Medizinisch adaptive Kompressionsbandagen (MAK), die die Haut weniger traumatisieren, aber dennoch die medizinisch bei CVI unbedingt empfohlene Kompressionstherapie gewährleisten, könnten hier Alternativen darstellen.^16^ Insbesondere unter okklusiven und/oder adhäsiven Wundverbänden entsteht ein feucht‐warmes Milieu. Dies kann vor allem bei längerer Tragedauer oder starkem Schwitzen zu Mazerationen führen sowie Reizungen oder (Mikro‐)Traumatisierung begünstigen. Somit sollte bei den Betroffenen die Verbandstrategie entsprechend überdacht und angepasst werden. Eingesetzt werden können insbesondere nicht‐adhäsive Wundprodukte, die möglichst atmungsaktiv sind und ein gutes Exsudatmanagement gewährleisten. Zudem wird empfohlen, die Verbandwechsel in kürzeren Intervallen, eventuell sogar täglich durchzuführen.^17^

Die EPDL zeigt meist einen chronisch‐rezidivierenden Verlauf. Bei adäquater Behandlung kann es zur Abheilung ohne Ulzeration kommen, jedoch bleibt die Haut atroph und anfällig für Rezidive. Unbehandelt beziehungsweise insuffizient therapiert drohen Ulzera, bakterielle Superinfektionen und in seltenen Fällen auch Plattenepithelkarzinome auf chronisch geschädigter Haut.^18^ Langfristig ist neben der konsequenten Hautpflege auch die Edukation der Betroffenen beziehungsweise Angehörigen und Pflegedienste wichtig, um ein Verständnis für das interdisziplinär und interprofessionell relevante Krankheitsbild zu schaffen.^19^

## DISKUSSION

Die derzeit sehr begrenzte Wissenslage zur EPDL führt häufig zu Fehldiagnosen und inadäquaten Therapieansätzen, was den Krankheitsverlauf unnötig verzögert und die Lebensqualität der Betroffenen erheblich beeinträchtigt. Es gibt bislang keine kontrollierten klinischen Studien zu dieser Krankheit. Meist handelt es sich bei den wenigen verfügbaren Publikationen um Kaustiken oder Fallserienberichte. In einer multizentrischen französischen Studie wurden die Daten von 36 Patienten mit EPDL aus 13 Behandlungszentren ausgewertet. Das durchschnittliche Alter lag bei 79,6 Jahren. Männer waren etwa fünfmal häufiger betroffen als Frauen; bei 91,7 % der Patientinnen lag eine CVI vor. Bei 53 % der Fälle traten die Hautveränderungen beidseitig auf, wobei die ventralen mittleren Drittel der Unterschenkel als häufigste Prädilektionsstellen identifiziert wurden. Eine vollständige Abheilung wurde bei knapp 80 % der Betroffenen nach durchschnittlich 2,5 Monaten beobachtet. In 97,3 % der Fälle kamen dabei topische Glukokortikoide zum Einsatz. Während der Nachbeobachtung traten bei fast 40 % der Patienten Rezidive auf, im Mittel nach 2,5 Monaten.^5^ In einer weiteren Studie wurde bei 24 von 400 Patienten mit fortgeschrittener CVI Pusteln an den Beinen dokumentiert. Bei 13 dieser 24 Patienten konnte eine Pilzinfektion nachgewiesen werden. Die Autoren diskutierten, dass die EPDL bei Patienten mit CVI unter Kompressionstherapie häufiger vorkommt, insbesondere dann, wenn eine Infektion mit Pilzen vorliegt.^4^ In einer retrospektiven Analyse von 16 Patienten mit einem Durchschnittsalter von 81 Jahren betraf die EPDL erneut etwa 5‐mal mehr Männer als Frauen. Die Prädilektionsstellen waren auch hier die mittleren Drittel der ventralen Unterschenkel, bei 63 % der Patienten beidseits. Klinisch wurden meist ockerfarbene Dermatitis und Hautatrophie beschrieben. Die Autoren diskutierten neben CVI auch UV‐bedingte Hautatrophie als potentiell begünstigenden Faktor. Therapeutisch waren topische Glukokortikoide in 12 von 13 Fällen bei einer mittleren Behandlungsdauer von sechs Monaten wirksam. Im Verlauf kam es bei 50 % der Patienten zu einem Rezidiv.^18^ In Italien wurden über einen Zeitraum von zehn Jahren 51 Patienten mit einem durchschnittlichen Lebensalter von 81 Jahren in einer Fallsammlung dokumentiert. Die Geschlechterverteilung war etwa ausgewogen. Bei 43 % der Betroffenen bestanden die Hautveränderungen beidseitig. Von den 40 Patienten, die in Hinblick auf eine atopische Diathese untersucht wurden, konnte diese bei 17 Betroffenen bestätigt werden.^6^ Auf der Grundlage der Untersuchung von drei Patienten mit EPDL wurde diskutiert, dass es sinnvoll sein könnte, Zinkglukonat zu substituieren, da bei diesen Patienten ein Zinkmangel nachgewiesen wurde.^20^

Möglicherweise kann die EDPL zur Gruppe der neutrophilen Dermatosen gezählt werden. Neutrophile Dermatosen sind eine heterogene Gruppe nicht‐infektiöser inflammatorischer Hauterkrankungen, die histopathologisch durch sterile Infiltration der Haut mit neutrophilen Granulozyten gekennzeichnet sind. Sie resultieren aus der Dysregulation des angeborenen, teils auch des adaptiven Immunsystems, die über eine Kaskade proinflammatorischer Zytokine zu überschießender Aktivierung und Rekrutierung neutrophiler Granulozyten führt und Inflammasome aktiviert. Neutrophile Dermatosen können vielfältige kutane und extrakutane Manifestationen verursachen und so mit erheblicher Morbidität und Mortalität einhergehen.^10^, ^21^ Sie können idiopathisch oder bei systemischen Erkrankungen wie Autoimmunerkrankungen, Neoplasien oder Infektionen auftreten oder ein Teilsymptom autoinflammatorischer Syndrome wie PAPA (Pyogene Arthritis, Pyoderma gangraenosum, Akne), PASH (Pyoderma gangraenosum, Akne, Hidradenitis suppurativa), DIRA (Defizienz des Interleukin‐1‐Rezeptor‐Antagonisten) oder CAPS (Cryopyrin‐assoziierte periodische Syndrome) sein.^22^ Bei diesen Patienten sollten daher immer, orientiert an der Anamnese und den klinischen Untersuchungsbefunden, weiterführende Untersuchung wie Differentialblutbild und apparative Bildgebung erfolgen.^8^, ^23^, ^24^ Klinisch gibt es in den initialen Stadien oft Gemeinsamkeiten der EPDL mit dem Pyoderma gangraenosum (PG), da beide Dermatosen mit sterilen Pusteln an den Streckseiten der Unterschenkel beginnen können. Hier ist eine Biopsie oft hilfreich, die beim PG im Gegensatz zu EPDL ein deutlich tieferes und dichteres Infiltrat mit neutrophilen Granulozyten und meist auch eine begleitende leukozytoklastische Vaskulitis zeigt. Dadurch resultieren bei PG im Gegensatz zu EPL fast immer sehr starke Schmerzen und ein dunkel‐livider Randsaum.^10^ Wenn oberflächliche (subcorneale) lokalisierte Pustulosen auftreten werden diese aktuell mit unterschiedlichen Bezeichnungen wie beispielsweise als akute lokalisierte exanthematische Pustulose (ALEP) klassifiziert.^25^, ^26^ Meist wird hierbei ein pathophysiologischer Zusammenhang mit (paradoxen) Medikamentenreaktionen diskutiert. Im klinischen Alltag gibt es immer wieder Patienten, deren Erkrankung nicht eindeutig einer bestimmten neutrophilen Dermatose zugeordnet werden kann. Für die Betroffenen sind jedoch vor allem die Identifikation relevanter auslösender Faktoren, gezielte Therapie und, soweit möglich, präventive Maßnahmen von zentraler Bedeutung.

Aufgrund der charakteristischen klinischen Präsentation, der vergleichbaren histopathologischen Befunde sowie der Hinweise auf zugrunde liegende pathophysiologische Mechanismen schlagen wir vor, die EPDL künftig als neutrophile Dermatose einzuordnen.

## FAZIT

Die EPDL ist eine idiopathische inflammatorische Dermatose mit besonderer topografischer Ausprägung, die selten diagnostiziert wird, aber gerade bei älteren Menschen und insbesondere bei Patienten mit CVI eine wichtige Krankheit ist. Aufgrund der Ähnlichkeit mit infektiösen oder autoimmunen Erkrankungen ist eine sorgfältige Differentialdiagnostik erforderlich. Die komplexen Therapien sind meist gut wirksam, erfordern jedoch ein konsequentes Hautpflegeregime sowie ein langfristiges Management in einem interdisziplinären und interprofessionellen Team.

## DANKSAGUNG

Open access Veröffentlichung ermöglicht und organisiert durch Projekt DEAL.

## INTERESSENKONFLIKT

Keiner.

## References

[1] Burton JL . Case for diagnosis. Pustular dermatosis of scalp. Br J Dermatol. 1977;97(Suppl. 15):67‐69. 884065 10.1111/j.1365-2133.1977.tb14339.x

[2] Lanigan S , Cotterill J . Erosive pustular dermatosis ‐ a common development in atrophic skin. Br J Dermatol. 1987;117:15.

[3] Bhargava S , Yumeen S , Henebeng E , Kroumpouzos G . Erosive pustular dermatosis: delving into etiopathogenesis and management. Life. 2022;12(12):2097. 36556462 10.3390/life12122097 PMC9784138

[4] Dawn G , Loney M , Zamiri M , et al. Erosive pustular dermatosis of the leg associated with compression bandaging and fungal infection. Br J Dermatol. 2003;148(3):489‐492. 12653740 10.1046/j.1365-2133.2003.05094.x

[5] Nicol P , Perceau G , Barbe C , et al. Erosive pustular dermatosis of the leg: a prospective, multicentre, observational study of 36 cases. Ann Dermatol Venereol. 2017;144(10):582‐588. 28532589 10.1016/j.annder.2017.02.007

[6] Di Altobrando A , Patrizi A , Vara G , Bianchi T . A 10‐year single centre experience of erosive pustular dermatosis of the leg: a possible correlation with atopic dermatitis? Dermatology. 2021;237(4):565‐567. 33477137 10.1159/000513352

[7] Brouard MC , Prins C , Chavaz P , et al. Erosive pustular dermatosis of the leg: report of three cases. Br J Dermatol. 2002;147(4):765‐769. 12366427 10.1046/j.1365-2133.2002.04878.x

[8] Chen Y , Yang S , Liu Y , Liu H , Luo Y . Scalp pustule as a manifestation of erlotinib‐induced skin toxicity: report of two cases and literature review. Clin Cosmet Investig Dermatol. 2025;18:1011‐1017. 10.2147/CCID.S511116 PMC12049118 40322509

[9] Bonnekoh H , Erpenbeck L . Neutrophile Dermatosen ‐ Pathomechanistische Konzepte und therapeutische Entwicklungen. J Dtsch Dermatol Ges. 2023;21(4):374‐380. 10.1111/ddg.15055_g 37070516

[10] Dissemond J , Moelleken M , Tasdogan A . Pyoderma gangraenosum: Pathogenese, Diagnostik und Therapie. Dermatologie. 2025;76(7):449‐458. 40549162 10.1007/s00105-025-05522-z

[11] Rock KL , Latz E , Ontiveros F , Kono H . The sterile inflammatory response. Annu Rev Immunol. 2010;28:321‐342. 20307211 10.1146/annurev-immunol-030409-101311 PMC4315152

[12] Luger T , Loske KD , Elsner P , et al. Topische Dermatotherapie mit Glukokortokoiden ‐ Therapeutischer Index. J Dtsch Dermatol Ges. 2004;2(7):629‐634. 16281629 10.1046/j.1439-0353.2004.03626.x

[13] Dall'Olio E , Rosina P , Girolomoni G . Erosive pustular dermatosis of the leg: long‐term control with topical tacrolimus. Australas J Dermatol. 2011;52(1):15‐17. 10.1111/j.1440-0960.2009.00584.x 21332681

[14] Cramer N , Wellmann P , Schön MP , Mössner R . Successful treatment of palmoplantar pustulosis with topical ruxolitinib. J Dtsch Dermatol Ges in press. 10.1111/ddg.15854 PMC12697319 40847898

[15] Dissemond J , Augustin M , Eming SA , et al. Modern wound care ‐ practical aspects of non‐interventional topical treatment of patients with chronic wounds. J Dtsch Dermatol Ges. 2014;12(7):541‐554. 24813380 10.1111/ddg.12351

[16] Dissemond J , Protz K , Stücker M . Kompressionstherapie in der Dermatologie. J Dtsch Dermatol Ges. 2023;21(9):1003‐1020. 10.1111/ddg.15161_g 37700410

[17] Dissemond J , Assenheimer B , Gerber V , et al. Lokaltherapie chronischer Wunden: Das M.O.I.S.T. Konzept. Dtsch Med Wochenschr. 2023;148(7):400‐405. 36940691 10.1055/a-1987-4999

[18] Wantz M , Perceau G , Goeldel AL , et al. [Erosive pustular dermatosis of the legs: retrospective study of 16 cases]. Ann Dermatol Venereol. 2011;138(2):93‐99. 21333818 10.1016/j.annder.2011.01.003

[19] Lichterfeld A , Hauss A , Surber C , et al. Evidence‐based skin care: a systematic literature review and the development of a basic skin care algorithm. J Wound Ostomy Continence Nurs. 2015;42(5):501‐524. 26165590 10.1097/WON.0000000000000162

[20] Salavert M , Franck F , Amarger S , et al. [Erosive pustular dermatosis of the leg: role of zinc deficiency?]. Ann Dermatol Venereol. 2006;133(12):975‐978. 17185927 10.1016/s0151-9638(06)71081-x

[21] Weiss EH , Ko CJ , Leung TH , et al. Neutrophilic dermatoses: a clinical update. Curr Dermatol Rep. 2022;11(2):89‐102. 35310367 10.1007/s13671-022-00355-8 PMC8924564

[22] Marzano AV , Damiani G , Genovese G , Gattorno M . A dermatologic perspective on autoinflammatory diseases. Clin Exp Rheumatol. 2018;36(Suppl. 110):32‐38. 29742056

[23] Erdmann M , Kiesewetter F , Schuler G , Schultz E . Erosive pustular dermatosis of the leg in a patient with ankylosing spondylitis: neutrophilic dysfunction as a common etiological factor? Int J Dermatol. 2009;48(5):513‐515. 19416383 10.1111/j.1365-4632.2009.03312.x

[24] Semkova K , Tchernev G , Wollina U . Erosive pustular dermatosis (chronic atrophic dermatosis of the scalp and extremities). Clin Cosmet Investig Dermatol. 2013;6:177‐182. 10.2147/CCID.S47019 PMC3712665 23874115

[25] Safa I , Ines L , Noureddine L , et al. Acute localized exanthematous pustulosis: Clinical features, pathophysiology, and therapy. Dermatol Ther. 2021;34(5):15087. 10.1111/dth.15087 34351040

[26] Tiong WS , Leong S , Chong WS . Acute localized exanthematous pustulosis of the leg. JEADV Clin Pract. 2023;2:937‐939.

